# Complete chloroplast genome sequence of *Pinellia ternata* (Thunb.) Breit, a medicinal plants to China

**DOI:** 10.1080/23802359.2020.1765207

**Published:** 2020-05-18

**Authors:** Ziping Cai, Hongxia Wang, Guoxiang Wang

**Affiliations:** a Institute of Chinese Herbal Medical, Gansu Academy of Agricultural Sciences, Gansu, Lanzhou, People’s Republic of China; b Engineering Laboratory of Germplasm Improvement and Quality Control of Gansu Province, Gansu, Lanzhou, People’s Republic of China

**Keywords:** Chloroplast genome, phylogenetic analysis, *Pinellia ternata* (Thunb.) Breit

## Abstract

*Pinellia ternata* (Thunb.) Breit is one of the commonly used traditional Chinese medicine with tuber as medicine. We report herein the complete chloroplast genome sequence of *Pinellia ternata* (Thunb.) Breit. It is length of 167,280 bp, which contained a small single-copy (SSC) region of 23,618 bp and a large single-copy (LSC) region of 92,450 bp, separated by two copies of an inverted repeat (IR) of 25,606 bp. The chloroplast genome contains 113 unique genes, including 79 PCG, 4 rRNA genes, and 30 tRNA genes. In addition, 19 genes contained one or two introns, which of those including 13 PCG genes possess a single intron and 2 PCG genes harbor two introns; and 6 tRNA genes harbor a single intron. In this study, *Pinellia ternata* is sister to *Pinellia pedatisecta* and clustered within the group consisting of the species that belong to Araceae.

*Pinellia ternata* (Thunb.) Breit is one of the commonly used traditional Chinese medicine with tuber as medicine (Zhao L et al. 1990; Guo et al. 2001; Xu et al. 2018), especially in the northwest region *Pinellia ternata* has high quality by the pharmaceutical industry (Wei 2009). The root tuber of *Pinellia ternata* has been used over the years in traditional medicine to treat myriad of diseases. Medicinal plants are widely used for the treatment of human diseases. There is a growing interest in *Pinellia ternata.* However, with the increase of the amount of *Pinellia ternata* maket demand, the wild resources are gradually exhausted. *Pinellia ternata* is a high economic value for exploitation and utilization wild herb medicine species with medicinal values in northwest China. The data of complete chloroplast genome will serve as a foundation for species identification, germplasm diversity, genetic engineering of *Pinellia ternata.* In this study, we aim to establish and characterize the complete chloroplast (cp) genome of *Pinellia ternata* and provide additional effective data for the phylogenetic study of Araceae in the future.

In this study, fresh leaves of *Pinellia ternata* (Thunb.) Breit were collected from solar greenhouse of Gansu Academy of Agricultural Sciences of China, located at 103.6848 E, 36.1001 N. The voucher specimen (No. HM1912181) was deposited at the Engineering Laboratory of Germplasm Improvement and Quality Control of Gansu Province, Lanzhou, Gansu, China; the procedure of DNA isolation, genome sequencing, and data processing all follow the previous research (Fang Yan et al. 2019).

The complete chloroplast genome was annotated with *Pinellia pedatisecta* (MN046890) as reference and has been submitted to GenBank with the accession number of MT193722. The chloroplast genome of *Pinellia ternata* is a circular quadripartite structure similar to major angiosperms chloroplast genomes (Hahn C et al. 2013). It is length of 167,280 bp, which contained a small single-copy (SSC) region of 23,618 bp and a large single-copy (LSC) region of 92,450 bp, separated by two copies of an inverted repeat (IR) of 25,606 bp.

The chloroplast genome contains 113 unique genes, including 79 PCG, 4 rRNA genes, and 30 tRNA genes. In addition, 19 genes contained one or two introns, which of those including 13 PCG genes (*atpF, ndhA, ndhB, petB, petD, rpl2, rpl16, rpoC1, rps12, rps16,* and *ycf68*) possess a single intron and 2 PCG genes (*clpP, ycf3*) harbor two introns; and 6 tRNA genes (*tRNA-Ala*(UGC), *tRNA-Gly*(UCC), *tRNA-Ile*(GAU), *tRNA-Leu*(UAA), *tRNA-Lys*(UUU), and *tRNA-Val*(UAC)) harbor a single intron.

The Bayesian phylogenetic tree was generated using TOPALi v2.5 (Milne et al. 2009) based on the complete chloroplast genome of *Pinellia ternata* and 15 other species from Araceae. Further, the phylogenetic tree analysis showed that *Pinellia ternata* was closely related to the genus *Pinellia pedatisecta* (Figure 1).

**Figure 1.**
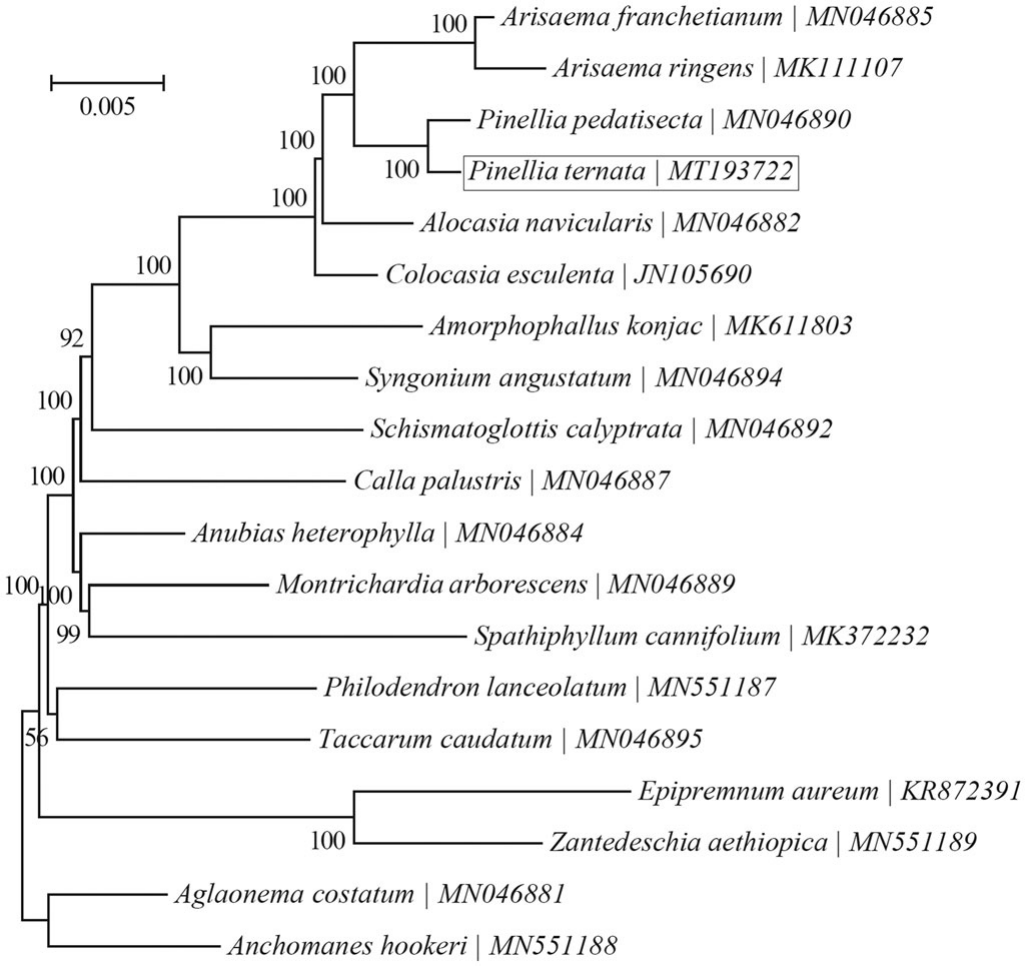
The phylogenetic tree (Bayesian inference) of *Pinellia ternata* (Thunb.) Breit and its related relatives based on the complete chloroplast genome sequences.

In this study, *Pinellia ternata* is sister to *Pinellia pedatisecta* and clustered within the group consisting of the species that belong to Araceae, and the determination of the complete plastid genome sequences provided a useful resource for new molecular data to illuminate the *Pinellia ternata* evolution.

## Data Availability

We confirm that the data supporting the findings of this study are available within the article and its supplementary materials. And the data that support the findings of this study has been deposited in GenBank (https://www.ncbi.nlm.nih.gov/) with accession numbers MT193722.
